# Analysis of Postural Control following Anterior Cruciate Ligament Reconstruction with Ipsilateral Peroneus Longus Tendon Graft

**DOI:** 10.5704/MOJ.2303.016

**Published:** 2023-03

**Authors:** PK Sahoo, MM Sahu

**Affiliations:** 1 Department of Physical Medicine and Rehabilitation, Swami Vivekanand National Institute of Rehabilitation Training and Research, Cuttack, India; 2 Department of Physiotherapy, Swami Vivekanand National Institute of Rehabilitation Training and Research, Cuttack, India

**Keywords:** anterior cruciate ligament, peroneus longus tendon, centre of pressure, postural control, force plate

## Abstract

**Introduction:**

Harvesting peroneus longus for ACL reconstruction is thought to create ankle instability which could add to postural instability from an ACL injury. This apprehension prevents its use as a graft of primary choice for many surgeons. To date, there is no evidence available describing changes in postural control after its use in ACL reconstruction. The purpose of the study was to analyse the changes in postural control in the form of static and dynamic body balance after ACL reconstruction with Peroneus Longus Tendon Graft and compare it with the unaffected limb at different time intervals.

**Materials and methods:**

Thirty-one participants with ACL injury were selected and subjected to an assessment of static and dynamic balance before and after ACL reconstruction using the HUMAC balance system. Outcome measures for Centre of Pressure (COP) assessment were average velocity, path length, stability score, and time on target. Comparison of scores was done pre-operatively as well as at three- and six-months post-reconstruction with Peroneus longus tendon graft.

**Results:**

Static balance of the affected limb showed significant improvement with a decrease in average velocity (F=4.522, p=0.026), path length (F=4.592: p=0.024) and improvement of stability score (F=8.283, p=0.001). Dynamic balance measured by the time on the target variable also showed significant improvement at six-month follow-up (F=10.497: p=0.000). There was no significant difference between the affected and non-affected limb when compared at the different time intervals.

**Conclusion:**

The static and dynamic balance, which is impaired after ACL injury, improves with ACL reconstruction with PLT autologous graft. Hence PLTG can be safely used as a graft for ACL reconstruction without affecting postural control and body balance.

## Introduction

The Anterior Cruciate Ligament Reconstruction (ACLR), is one of the common ligament reconstruction procedures done in the knee that restores the joint stability, allowing for a faster return to pre-injury function^1^. Hamstrings and patellar tendons are the preferred graft options for ACL reconstruction, each with its advantages and disadvantages^2-3^. Extensive research has been done to determine the optimum graft; however, the results are still up for debate^2^. Recently full-thickness Peroneus Longus Tendon (PLT) has been used as an alternative autograft option for ACL reconstruction with functional outcomes comparable to hamstring tendon autografts4. Biomechanically it is as strong as the native ACL5 and even superior to hamstring graft^6^. It has been considered as a safe and effective alternative for reconstruction of ACL in non-athletic patients. However, controversies continue to exist about donor ankle morbidity after the PLT graft (PLTG) harvest. Some authors claim that at least in the first year after harvesting the complete length PLTG, there was a transverse plane balance deficit around the donor ankle and hindfoot. They even recommend that PLT autograft be used only in reconstructive surgeries for multi-ligament injuries after all other graft choices have been exhausted^7^. Many studies subsequently looked at donor ankle function following PLTG harvest for ACL reconstruction and found satisfactory outcomes, although their conclusions were based on parameters such as functional score and peroneal strength^4^. Even then, there is a paucity of research on other essential aspects of donor site morbidity such as gait and balance. This is most likely the reason why PLTG is not widely recognised. Further many of the studies come from the East^4^.

The decision to return to play is heavily influenced by postural stability and balance parameters^8^. Postural control is described as an individual’s ability to maintain body stability and orientation while controlling their body position in space. It necessitates complex sensory-motor integration. The ACL is rich in mechanoreceptors and serves as one of the somatosensory organs for whole-body postural regulation9. Although ACL instability is a peripheral musculoskeletal problem, it is now recognised as a proprioceptive neurophysiological dysfunction^10-11^. Patients with ACL injuries have been found to have impaired postural stability and to have a proprioception deficit^11-12^. Persistent postural impairments were also observed following ACL reconstruction^13-14^, which has become a major cause of disability^15^. Regardless of the graft used, rehabilitation objectives following ACL reconstruction should focus on improving postural stability by facilitating neuromuscular control^15,16^.

The Peroneus longus muscle plays an important function in both active as well as passive stabilisation of the ankle and hindfoot stability^17-18^. As a result, it is assumed that PLTG harvest for ACL reconstruction might affect the already compromised body balance by influencing postural stability at the donor ankle site^7^. This apprehension prevents its use as a graft of primary choice for many surgeons. However, the synergistic action of the intact Peroneus Brevis muscle is likely to restore the postural stability of the donor ankle to some extent^4,6^. Biomechanical evidence suggests that Peroneus brevis dominates among the peronei muscles and Peroneus longus act as an accessory evertor of ankle joint complex^19^. Hence it could be assumed that harvesting PLTG with intact Peroneus brevis musculature would either adequately substitute the PLT or its synergistic function would restore the function of the donor ankle function to a an acceptable level, justifying its harvest for graft option^19^. However, no study has been done showing prospective changes in postural stability after harvesting autologous PLTG for ACL reconstruction.

We did a thorough search on PubMed using MeSH terms such as “Anterior cruciate ligament reconstruction”, “peroneus longus tendon graft”, “centre of pressure”, “postural control”, and “balance”. No researchers investigated postural control in patients who had ACL reconstruction with peroneus longus autograft. There was no evidence available comparing postural regulation and stability in ACL reconstruction patients with PLT grafts before and after ACL reconstruction. We did not find any study, assessing postural balance following ACL reconstruction using any other graft too. So, this is a novel study with an endeavour of analysing and comparing the change in postural stability after an ACL reconstruction with PLTG. Harvesting peroneus longus is thought to create ankle instability which could add to postural instability from an ACL injury. This apprehension prevents its use as a graft of primary choice for many surgeons. The purpose of the study was to analyse the changes in postural control in the form of static and dynamic body balance after ACL reconstruction with PLTG and compare it with the unaffected limb at different time intervals. We hypothesised that ACL rupture and subsequent harvesting of PLT for surgical reconstruction would affect both static and dynamic body balance.

## Materials and Methods

The current study was a prospective analytic research design conducted at a national rehabilitation institute in India for 24 months, from March 2019 to March 2021. The study has been approved by the ethical committee of the Institution. It was performed conforming with the Declaration of Helsinki and informed consent was taken from all the participants. Of a total of 44 subjects who reported to the out-patient department for appreciable knee instability, 31 cases met the inclusion criteria and were recruited for the study.

Inclusion criteria were: (i) Unilateral isolated ACL tear with no concomitant tear of other ligaments of the knee, (ii) no evidence of meniscal repair, (iii) no history of trauma or surgery to the opposite knee, (iv) no evidence of any systemic problems affecting the posture and gait of the subject.

Exclusion criteria were: (i) arthroscopically confirmed chondral lesions, (ii) visible mal alignments in the leg, (iii) any acute or chronic inflammation of the joints, (iv) pre-existing ankle injury or ankle instability, (v) any general systemic or mental illness. Patients were also excluded if they could not perform the postural stability test due to pain or limited knee joint motion.

All the participants were evaluated clinically for instability, to diagnose the grade III ACL tear. This was later confirmed by magnetic resonance imaging (MRI). All the selected subjects had undergone ACL reconstruction with autologous trifold peroneus longus tendon graft by a senior surgeon. The PLTG was harvested from the ipsilateral side with a 2cm incision at 1cm above and behind the lateral malleolus. The free distal end of the peroneus longus tendon was tenodesized with the nearby peroneus brevis tendon. Single bundle ACL reconstruction with PLTG was done by a standard arthroscopic method. Non-articulated long knee brace was used during immediate post-operative period. Since both quadriceps and hamstrings were intact in this procedure, a gold standard accelerated rehab protocol was prescribed to facilitate the knee function. The protocol was tailored to a Home-based rehabilitation program with addition of exercises to improve ankle range of movement, peronei strength, proprioception, and balance with reference to ankle joint complex. All the subjects received an educational pamphlet outlining the therapeutic procedure, as well as a pictorial illustration of important exercises. They were instructed to report every three weeks for review and supervision of therapy. The principal evaluator recorded the outcome measures before surgery and at the three-month and six-month post-ACL reconstruction follow-ups.

We used the Humac Balance system [HUMAC2015® Version: 15.000.0103 © Computer Sports Medicine, Inc.] (www.csmisolutions.com) for the balance assessment. Both static and dynamic balance of the body can be measured on a force plate using a dedicated software of the system. The balance performance of the individual is enhanced by the attached visual feedback system20. The results are consistent and highly reproducible having an acceptable error of up to 0.18%^21^. In Humac, the centre of pressure (COP) provides objective data of postural competence^21^ COP is the point of applying resultant force in the vertical z-axis acting on the base of support. It is the most used parameter to evaluate postural balance using a force plate^22-23^. The Humac force plate provides information about spatial and temporal alterations of body position to maintain balance on the horizontal and vertical axis.

Outcome measures used for our study included evaluation of static balance by the measurement of bilateral and unilateral centre of pressure (COP). Stability Score (%), Path Length (cm), and Average Velocity (cm/s) were taken as study variables. Path length denotes the average displacement of COP from the centre position and Average velocity measures the displacement of COP data points per unit of time^21^. The variable for dynamic balance was Time on Target (%) of the mobility dimension of the Humac balance system.

**Fig. 1:**
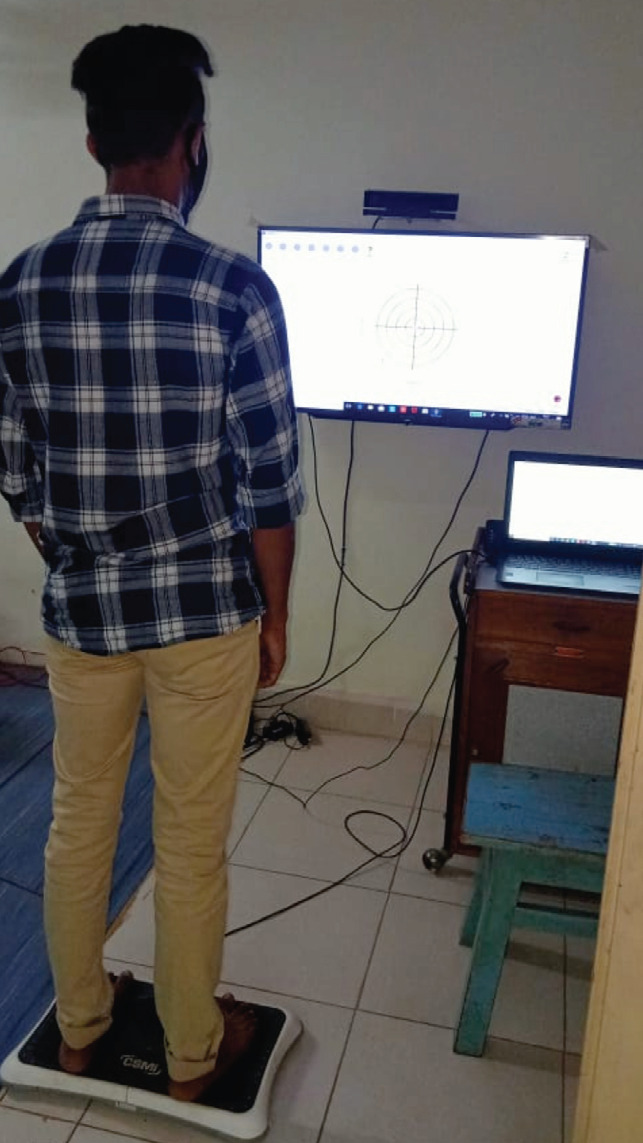
Double leg standing postural control assessment on HUMAC balance system.

**Fig. 2:**
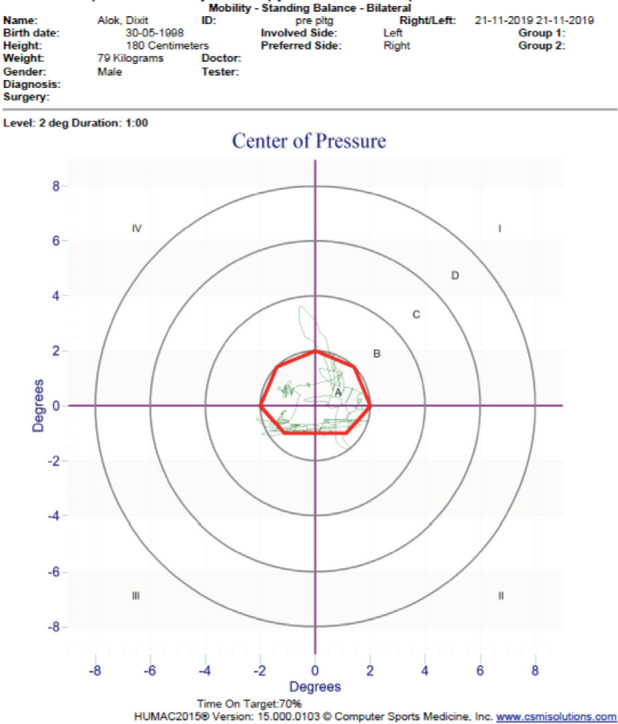
Time on target for assessment of dynamic stability.

**Fig. 3:**
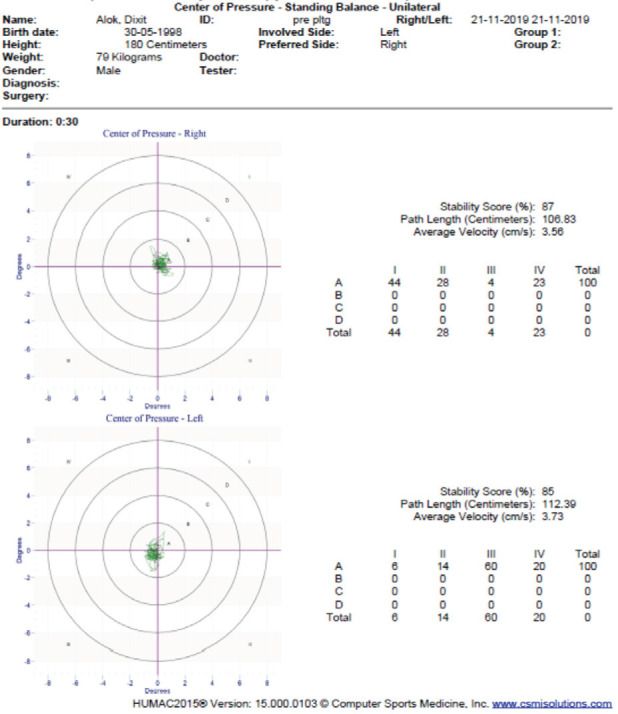
COP parameters for static stability assessment.

Method of Assessment: Postural stability in the Humac Balance system was assessed as per the procedure described in the author's previous article^20^. The participants were advised to stand barefoot atop the force plate with arms at the side, eyes open, and fixed on the magenta on the display monitor for both bilateral and unilateral standing balance measurements (Fig.1). For dynamic balance measurement, the subjects were asked to follow a moving target on the display board (Fig. 2). The set parameters were COP (bilateral and unilateral) each for 30 seconds, and mobility, at level two for one minute^20^. The participants were first familiarised with a trial round, then the best trial of three subsequent measurements was recorded (Fig. 3).

The Statistical package for Social Science (SPSS) version 18.0 was used for statistical analysis. The normality of data was confirmed using Shapiro-Wilk’s test, and appropriate parametric statistics were computed. One Way ANOVA was used to compare Bilateral, Affected and Sound groups at different time intervals. Pairwise comparisons were made using post-hoc Tukey’s test. Repeated measure ANOVA was employed to compare study groups at various study visits (pre-op, three month and six-month post-ACL reconstruction). Wilk’s Lambda was calculated, followed by appropriate Mauchly’s test of sphericity and Pairwise comparisons between study visits were done after Bonferroni adjustment for multiple comparisons. A p<0.05 was considered significant for all statistical inferences.

## Results

During the study period, out of 44 patients with ACL reconstruction, 31 patients with a mean age of 28.42+8.932 years conformed with the inclusion criteria. Their demographic characteristic is shown in (Table I). All the study participants were clinically right dominant.

**Table I:** Demography.

Variable	Mean	Std. Deviation	Std. Error
Age (year)	28.42	8.932	1.604
Height (cm)	1.68	0.066	0.0119
Weight (kg)	68.45	12.011	2.157
BMI	24.2692	4.410	0.792
	**Category**	**Frequency**	**Percent**
Gender	Female	3	9.68
	Male	28	90.32
Affected side	Left	13	41.94
	Right	18	58.06
Dominant Side	Left	0	0
	Right	31	100

As per the HUMAC guideline, observation of a greater stability score, and a decrement in the path length and average velocity indicates improvement of static balance and increment in time on target score indicates improvement of dynamic balance^22^.

The average velocity score showed statistically significant improvement during single leg standing balance on the affected leg only (F=4.522, p=0.026) (Table II). The significance was observed during the pair-wise comparison between pre- and six-month, and three-month and six-month post-op scores (p=0.016, p=0.009, respectively). But for bilateral or unilateral stance on the sound leg, the differences were not significant (Table II).

**Table II:** Repeated measure ANOVA among various study visits with pair wise group comparison.

Variable	Group	Pre (Mean±S.D.)	Three Month (Mean±S.D.)	Six Month (Mean±S.D.)	Wilks' Lambda	p	F	p
Stability score	Bilateral	87.65±14.011	91.45±6.324	93.29±3.111	0.827	.064	3.041	.079
	Affected	84.06±5.904^a^	86.84±3.813	88.16±2.296^b^	0.662	.003	8.283	.001
	Sound	84.94±5.790^a^	87.61±2.526^b^	87.65±3.147^b^	0.777	.026	7.133	.007
Path length	Bilateral	42.361±30.985^a^	30.680±12.877	26.905±8.599^b^	0.796	.036	5.331	.020
	Affected	109.859±39.727^a^	103.335±23.443^a^	93.641±23.602^b^	0.741	.013	4.592	.024
	Sound	108.422±33.287	100.601±22.415	96.195±22.854	0.885	.169	2.850	.009
Average Velocity	Bilateral	3.831±.14.897	1.023±.429	.895±.287	0.889	.180	1.148	.293
	Affected	3.660±1.326^a^	3.442±.780^a^	3.122±.788^b^	0.744	.014	4.522	.026
	Sound	3.603±1.115	3.349±.747	3.205±.764	0.890	.184	2.722	.098
Time target		90.10±9.123^a^	93.45±6.516^a^	95.77±5.340^b^	0.578	.000	10.497	.001

Notes: (S.D.- Standard Deviation)

The significance of difference over time was assessed by Repeated Measure ANOVA and Tukey’s multiple comparison (^a,b^ represent p<0.017)

Statistically, Path length improved significantly during bilateral (F=5.331, p=0.020) and unilateral standing balance on the affected leg (F=4.592: p=0.024). (Table II). The bilateral standing balance at the six-month post-op visit showed significant improvement as compared to its pre-operative score (p=0.014). For the balance of the affected leg, significance was observed during the pair-wise comparison between the pre- and six-month, and three-month and six-month post-op score (p=0.015, p=0.009, respectively). No significant difference was observed for the unaffected limbs during any visit (Table II).

The improvement in Stability score was statistically significant only for unilateral stance on both the affected and unaffected limbs (F=8.283, p=0.001: F=7.133, p=0.007, respectively) (Table II). Affected leg scores were significantly different at the six-month post-ACL reconstruction visit as compared with its pre-operative value (p=0.001) (Table III). For the standing balance of the unaffected leg, significant differences were found during pre- and six-month, and three-month and six-month comparisons (p=0.012, p=0.006) (Table III).

**Table III:** Comparative analysis of postural parameters at different time interval.

Dependant Variables	Time	Comparison	Mean Difference	Std. Error	p	95% Confidence Interval for Lower Bound	
						Lower Bound	Bound Bound
Bilateral stability score	pre	three months	-3.806	2.806	.185	-9.537	1.924
		six months	-5.645*	2.645	.041	-11.047	-.243
	three months	six months	-1.839	1.219	.142	-4.328	.651
Affected stability score	pre	three months	-2.774*	1.187	.026	-5.199	-.349
		six months	-4.097*	1.079	.001	-6.300	-1.893
	three months	six months	-1.323	.770	.096	-2.894	.249
Sound stability score	pre	three months	-2.677*	.912	.006	-4.539	-.816
		six months	-2.710*	1.009	.012	-4.770	-.649
	three months	six months	-.032	.431	.941	-.913	.848
Bilateral path length	pre	three months	11.682*	5.719	.050	.003	23.361
		six months	15.456*	5.899	.014	3.409	27.503
	three months	six months	3.774	2.363	.121	-1.051	8.599
Affected path length	pre	three months	6.524	5.938	.281	-5.603	18.650
		six months	16.217*	6.306	.015	3.338	29.097
	three months	six months	9.694*	3.462	.009	2.623	16.765
Sound path length	pre	three months	7.822	5.497	.165	-3.404	19.048
		six months	12.227	6.505	.070	-1.059	25.513
	three months	six months	4.405	2.860	.134	-1.436	10.247
Bilateral avg. velocity	pre	three months	2.808	2.681	.303	-2.668	8.283
		six months	2.935	2.681	.282	-2.540	8.411
	three months	six months	.128	.079	.117	-.034	.290
Affected avg. velocity	pre	three months	.218	.198	.281	-.188	.623
		six months	.538*	.211	.016	.107	.970
	three months	six months	.321*	.116	.009	.085	.557
Sound avg, velocity	pre	three months	.254	.183	.174	-.119	.627
		six months	.398	.217	.077	-.045	.842
	three months	six months	.144	.096	.143	-.051	.340
Time on target	pre	three months	-3.355*	1.457	.028	-6.331	-.379
		six months	-5.677*	1.342	.000	-8.418	-2.937
	three months	six months	-2.323*	.856	.011	-4.070	-.575

Notes: Based on estimated marginal means

*. The mean difference is significant at the .05 level.

b. Adjustment for multiple comparisons: Least Significant Difference (equivalent to no adjustments).

Dynamic balance measured by the time on the target variable also showed significant difference statistically during the pre- and six-month, and three-month and six-month comparisons (F=10.497: p < 0.000, p=0.001) (Table II).

There was no significant difference statistically in the group comparison of all these balance parameters, between affected and unaffected limb at different time intervals such as pre-operatively and at three-month and six-month post-ACL reconstruction follow-ups (Table IV).

**Table IV:** Comparison of balance parameters between sound and affected limb.

Variable	Study visit	Affected (Mean±S.D.)	Sound (Mean±S.D.)	Mean Difference	95% Confidence Interval		t	p
					Upper = Lower			
Stability score (%)	Pre	84.06±5.90	84.94±5.79	-0.871	-3.842	2.100	-0.586	.560
	Three Months	86.84±3.81	87.61±2.52	-0.774	-2.423	0.874	-0.942	.350
	Six Months	88.16±2.29	87.65±3.14	0.516	-0.886	1.918	0.738	.464
Path length (cm)	Pre	109.85±39.72	108.42±33.28	1.436	-17.195	20.068	0.154	.878
	Three Months	103.33±23.44	100.60±22.85	2.734	-8.918	14.387	0.469	.640
	Six Months	93.64±23.60	96.19±24.83	-2.553	-14.357	9.249	-0.433	.667
Average velocity (cm/sec)	Pre	3.660±1.32	3.603±1.11	0.056	-0.566	0.679	0.182	.856
	Three Months	3.442±0.78	3.349±0.74	0.093	-0.295	0.481	0.480	.633
	Six Months	3.122±0.78	3.205±0.76	-0.083	-0.477	.31124	-0.422	.675

Notes: The mean difference is significant at the .05 level

## Discussion

In comparison to the pre-operative score, all the means of balance variables improved gradually during bilateral as well as unilateral stance at different follow-ups in our study. At six months post-ACL reconstruction, the differences were statistically most significant for the unilateral stance on the affected side. At any time, interval, however, there was no significant difference between the affected and the unaffected limb.

Evidence highlighted the contribution of the neuro-sensory function of ACL in postural control^24^. Thus, a postural deficit is usually expected in the ACL injured limb, justifying ACL reconstruction^11-12^. Hamstring and other established grafts, that were used for ACL reconstruction have shown satisfactory outcomes^25^. Similar observations were obtained in our subjects after ACL reconstruction with PLTG. Following the ACL reconstruction with PLTG, a consistent improvement in the Means of all the balance variables suggested an improvement in their postural stability and overall body balance. It implies that even if the whole body or single-leg stability declines after an ACL injury, it can be restored back to an optimal level after ACL reconstruction with PLT graft in the course of time. Our findings were comparable to other studies on postural balance following ACL reconstruction with different grafts which demonstrates that the reconstructed limb improved after the operative repair^15-16^.

Usually, balance and postural stability are taken as the determining factors to consider the possibility of a safe return to activity and sports participation after ACL injury or ACL reconstruction^15^. As most sports activities involve a single leg stance, its importance is emphasised to assess postural control in patients with ACL reconstruction^13^. In comparison to the pre-operative values, our assessment of single-leg balance on the affected leg revealed a considerable improvement in stability score at six-month after ACL reconstruction. The Path length and velocity are the most used parameters of Humac and are also sensitive to detect any balance impairments^22,26^. Based on these scores, we observed that balance whilst standing on the affected leg improved with time and was significant at six-month after surgery. In the unaffected side, balance was not much influenced as compared to the affected side following ACL reconstruction. As a result, we assumed that the balance of the injured limb was more affected than the unaffected side, which could be attributable to neurophysiological dysfunction after an ACL injury or donor ankle morbidity following PLTG harvest. However, it steadily improved over time, and at six-month, it was on par with the healthy limb.

Though the scores for balance of the unaffected limb balance was low during the pre-operative period to establish limb symmetry and preserve total body balance, they were better than the score of the affected leg. Path length and velocity scores were not significantly affected, indicating fair stability in the unaffected leg. As a result, there was little room for improvement of balance in post-operative period for the unaffected side balance from an already high pre-operative value. Only the stability score, which was lower pre-operatively, improved quickly to reach its peak within three months and then stayed consistent after that. Our findings were congruous with those of Wiggins *et al* and Linard *et al* in prior research^15,27^. Tookuni *et al* also found that the single-leg balance was reduced on both the unaffected and operated sides, with the operated side having a significant effect. They did not, however, discover any effect of leg dominance on single leg balance^28^. Laboute *et al* observed bilateral kinaesthetic deficit in post-ACL reconstruction patients compared to the control group (p<0.001 and p=0.011), which was significantly higher on the operated side (p=0.001). They found a fast recovery on the unaffected side. They also noticed that the re-trained patients had no significant difference between the operated and uninjured knees^16^.

In our study, analysis of bilateral standing balance revealed a clinically linear improvement in all the COP variables for both static and dynamic body balance. The time on target score of dynamic balance, when compared at different time intervals, showed a statistically significant improvement at six-month post-ACL reconstruction as compared to the pre-operative and three-month score. The static body balance assessment, on the other hand, was almost uninfluenced. This supports the “Central Impairment Theory”^14,16^ which is a compensatory mechanism induced by the central neurophysiological system to preserve the symmetry between legs and body equilibrium following ACL injury or reconstruction^28-29^. According to Hoffmann *et al*, in the event of a unilateral ACL rupture, the body would restore symmetry by inhibiting the un-affected limb. Although overall postural control may be compromised following an ACL injury, leg symmetry can be restored^30^. In our research, we also noticed a similar pattern. Pre-operatively we observed a maintained static body balance with a reduction in the balance scores of both the unaffected and affected limb. Furthermore, when comparing affected and unaffected limbs at different time intervals, the variations in Postural parameters were very little and statistically insignificant. Our findings were consistent with the earlier studies. Researchers reported a reduction in static postural control in both affected and unaffected legs after ACL rupture^29^. Lehmann et al reported no variations in sway velocity between injured and non-injured ACL patients in a study^11^. Culvenor et al also noticed that the dynamic balancing performance, as measured by COP path velocity was lower than the controls, but it was similar between the reconstructed limb and the uninjured contralateral limb^14^. Thus, our data corroborated with the “Central Impairment Theory”. The centrally mediated postural control mechanism always tries to maintain the symmetry between the two legs to achieve overall body balance^10,16,30^.

In the current study, the postural control parameters in bilateral stance showed a significant improvement only in the path length score at six-month post-ACL reconstruction as compared to the pre-operative scores. It could be that the high pre-operative baseline score left no room for post-operative improvement. Be that as it may, since path length is a more sensitive variable, it only exhibited minor changes in overall body balance at six-month. Furthermore, bilateral standing represents the body's overall balance and is dependent on the postural stability of each leg. As they increased the most at six-month post-ACL reconstruction visit, the overall body balance was also significantly improved at this time.

There are some limitations to the current study. The comparison of gender effect on postural control following ACL reconstruction was limited due to the small number of female participants. After discharge from the hospital, the post-operative rehabilitation protocol was a home-based program that could not be supervised. Giving more attention to operated limbs by the subjects would have created a bias in our study. All patients were allowed to participate in sports activities after the last record of postural stability at six-month. However, long term studies are suggested to observe if the achieved postural stability is maintained.

## Conclusion

The study concluded that following ACL reconstruction with PLT autograft, participants improved their balance and postural control, which was impaired after ACL injury. Both static and dynamic body balance improved in the early stages after ACL reconstruction, but the improvement was significant at six-month only. Statistical comparisons between affected and unaffected limbs during the different study intervals did not show significant differences which led us to conclude that peroneus longus tendon autograft could be considered as an alternative for reconstructing the ACL without affecting overall postural control and balance of the body.
